# The Role of Long Non-Coding RNA in Rheumatoid Arthritis

**DOI:** 10.3390/ijms26020560

**Published:** 2025-01-10

**Authors:** Kajetan Kiełbowski, Maciej Ćmil, Wojciech Jerzy Biniek, Estera Bakinowska, Andrzej Pawlik

**Affiliations:** Department of Physiology, Pomeranian Medical University, 70-111 Szczecin, Poland; kajetan.kielbowski@onet.pl (K.K.); cmilmaciej@gmail.com (M.Ć.); wojciech.biniek6@gmail.com (W.J.B.); esterabakinowska@gmail.com (E.B.)

**Keywords:** rheumatoid arthritis, non-coding RNA, long non-coding RNA, inflammation

## Abstract

Rheumatoid arthritis (RA) is a chronic autoimmune disease that leads to joint damage and physical dysfunction. The pathogenesis of RA is highly complex, involving genetic, epigenetic, immune, and metabolic factors, among others. Over the years, research has highlighted the importance of non-coding RNAs (ncRNAs) in regulating gene expression. Given their dysregulation in numerous conditions, ncRNAs are thought to play a role in pathological processes. In RA, aberrant levels of circulating long ncRNAs (lncRNAs) are commonly observed in peripheral blood, along with their dysregulated expression in peripheral blood mononuclear cells and synovial tissue. This review discusses the involvement of lncRNAs in inflammation and the aggressive characteristics of fibroblast-like synoviocytes, a key cellular population driving RA progression.

## 1. Introduction

Rheumatoid arthritis (RA) is a chronic progressive autoimmune disease that leads to joint damage and physical dysfunction. The pathogenesis of RA is highly complex, with genetic, epigenetic, immune, and metabolic factors all contributing to its development [1]. Recently, much attention has been targeted toward the relationship between aging and autoimmunity. Accelerated biological aging has been recently associated with an increased risk of RA [2]. The condition occurs more frequently in females, with joint pain, swelling, and morning stiffness as characteristic symptoms [1]. In addition to these typical manifestations, extra-articular symptoms are closely associated with the clinical presentation of RA. These may include rheumatoid nodules and vasculitis, among other features [3]. Because of the systemic nature of RA, the disease course can affect other organ systems. For example, interstitial lung disease is considered one of the most common complications of RA. A recent meta-analysis reported that the pooled prevalence of RA-associated interstitial lung disease was approximately 19% [4].

In 2021, the prevalence of RA in Poland was 0.7% [5], while the global prevalence of the disease is estimated at approximately 0.5% [6]. Because an RA diagnosis is associated with reduced life expectancy and a high cost of therapeutics [7], there is ongoing discussion regarding potential preventive methods to reduce the risk of developing RA or to optimally suppress its progression at an early stage [8]. A deeper understanding of the nature of RA will provide essential insights for the development of preventive and advanced therapeutic strategies.

Chronic inflammation is a crucial component of RA. As previously mentioned, the disease involves interactions between immune and articular cells, with macrophages and T cells frequently highlighted as key contributors to heightened inflammatory responses. Specifically, macrophages are major producers of tumor necrosis factor-α (TNF-α), a cytokine now widely targeted by TNF-α inhibitors (biological disease-modifying antirheumatic drugs) such as adalimumab and infliximab [9]. Regarding T cells, various subtypes are involved in RA progression. An abnormal presence of these cells is associated with increased secretion of pro-inflammatory cytokines and chemokines, which further promotes immune cell infiltration and tissue damage. For example, T-helper 1 (Th1) and T-helper 17 (Th17) cells are extensively studied in the context of RA because of their association with disease activity [10,11,12]. Conversely, a reduced presence of regulatory T cells (Tregs), which secrete anti-inflammatory mediators, is observed in RA-affected joints [13]. Under these pathological conditions, fibroblast-like synoviocytes (FLSs) adopt an inflammatory and invasive phenotype. Over recent years, there has been growing interest in investigating non-coding RNA (ncRNA) in the pathogenesis of RA.

ncRNA is a broad family of RNA molecules that play a crucial role in maintaining homeostasis. The importance of these molecules was recently highlighted when the discovery of microRNAs (miRNAs) was recognized with the 2024 Nobel Prize in Medicine and Physiology [14,15]. miRNAs, along with long ncRNA (lncRNA) and circular RNA, are significant classes of ncRNA that regulate gene expression. For instance, miRNAs can bind to their target mRNA molecules, suppressing translation. Researchers have shown that the expression of these molecules is dysregulated in various diseases. Given their impact on gene expression, aberrant ncRNA expression contributes to the pathophysiology of numerous conditions. According to the recent consensus statement, ncRNAs are considered lncRNAs when they are over 500 nucleotides long [16]. They play crucial roles in regulating gene expression at various levels, including transcription, translation, and post-translational modifications [17,18]. With regard to protein-coding genes, lncRNAs can be categorized into antisense, intronic, and intergenic [16]. These molecules are involved in a wide range of biological processes, such as chromatin remodeling, transcription control, and post-transcriptional processing, and they have been implicated in the pathogenesis of several diseases, including autoimmune disorders [19]. lncRNAs can also influence immune cell function and are differentially expressed in various tissues, suggesting their role in specific organ damage in autoimmunity [18]. Furthermore, lncRNAs can regulate the secretion of cytokines, such as TNF-α, via nuclear factor kappa-B (NF-κB), which is crucial for inflammatory responses [20]. In RA, lncRNAs such as NEAT1, PVT1, ZFAS1, PICSAR, and GAPLINC regulate inflammatory responses and cell proliferation by interacting with miRNAs and other molecular pathways, highlighting their role in disease progression and potential as therapeutic targets [17,21,22,23,24]. In this review, we discuss the latest findings on the role of lncRNAs in the pathogenesis of RA, focusing on studies investigating their effect on inflammation and the aggressive features of FLSs.

## 2. Long Non-Coding RNA in the Pathogenesis of Rheumatoid Arthritis

### 2.1. Inflammation

By influencing gene expression, lncRNA molecules significantly contribute to the inflammatory status within affected joints. New studies frequently report additional molecules associated with inflammation. For instance, in a recent article by Chini et al., the authors used RNA sequencing to identify novel lncRNAs linked to inflammation in macrophages. Notably, the researchers demonstrated that some of the examined molecules depend on NF-κB activation [25]. This finding is significant because NF-κB is a transcription factor strongly associated with pro-inflammatory responses, such as the upregulation of pro-inflammatory cytokines. Understanding that NF-κB also influences the expression of ncRNA molecules further expands the inflammatory network of this transcription factor. Several lncRNAs have been studied in the context of immune responses in RA.

To begin with, the lncRNA nuclear paraspeckle assembly transcript 1 (NEAT1) is overexpressed in the synovial tissue of patients with RA [17,26]. Its expression in peripheral blood mononuclear cells (PBMCs) significantly correlates with clinical and diagnostic parameters in RA, such as the Disease Activity Score 28 with erythrocyte sedimentation rate (DAS28-ESR) and C-reactive protein (CRP) level [27]. Furthermore, NEAT1 expression decreases with treatment [22], further suggesting its involvement in disease pathogenesis and highlighting its potential as a marker of treatment response.

The mechanism of action of NEAT1 is closely linked to the inflammatory responses typical of RA pathophysiology. Specifically, NEAT1 connects NF-κB activation with the secretion of interleukin (IL)-1β and IL-6, as targeting this lncRNA with small interfering RNA (siRNA) significantly reduces the expression of phosphorylated NF-κB and the aforementioned cytokines [17]. Conversely, NF-κB also appears to influence NEAT1 expression, as p65 can bind to the NEAT1 promoter [28]. Other transcription factors involved in NEAT1 expression include p53, STAT3, E2F1, and HuR, among others, in various cell types [29]. Silencing NEAT1 in mice is associated with a reduced presence of T cells and macrophages in synovial tissues. Guo et al. demonstrated that NEAT1 is positively associated with IL-18, as overexpression of this lncRNA upregulated IL-18 in synovial cells [26]. IL-18, which is found at increased concentrations in the serum of patients with RA [30], plays a role in the pathogenesis of various autoimmune and inflammatory conditions. Interestingly, IL-18 can also amplify immune responses by T cells. The involvement of IL-18 in these mechanisms and diseases is well summarized in a review by Landy et al. [31].

**Figure 1:**
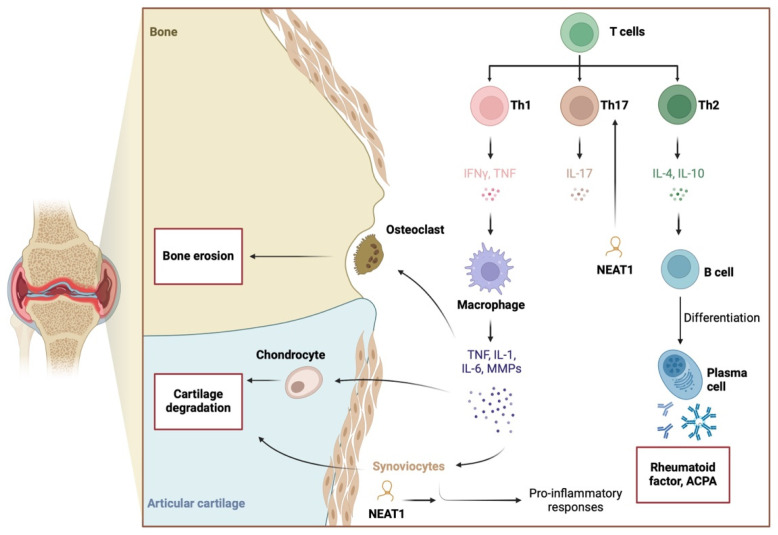
Pathogenesis of RA involves pro-inflammatory responses, to which NEAT1 is suggested to significantly contribute. The lncRNA stimulates the expression of pro-inflammatory cytokines and enhances the differentiation of Th17 cells. Created in BioRender. Kiełbowski, K. (2025) https://BioRender.com/c05i163.

NEAT1 activity is also suggested to influence T cell differentiation. Specifically, NEAT1 expression increases in differentiated Th17 cells compared to undifferentiated subtypes. Moreover, NEAT1 knockdown reduces Th17 cells and the expression of IL-17 [32] (Figure 1). Dysregulation of Th17/Treg populations is considered a key pathophysiological mechanism driving chronic inflammatory responses [10,33]. Mechanistically, NEAT1 interacts with STAT3 to promote Th17 differentiation [32]. STAT3 is part of the JAK/STAT pathway, which is targeted in patients with RA using approved JAK inhibitors. In other disease models, NEAT1 has been shown to regulate IL-17 expression, directly modulating Th17 responses [34]. Additionally, NEAT1 knockdown can increase the presence of Tregs and restore the dysregulated Th17/Treg ratio [35]. Introducing Tregs or methods aimed at expanding their population are among potential treatment strategies in RA [36].

In addition, NEAT1 is implicated in stress response mechanisms. A positive correlation was observed between NEAT1 and synoviolin, an E3 ubiquitin ligase involved in the process known as ER-associated protein degradation [37]. The precise role of synoviolin remains unclear, and conflicting findings have been reported. Inhibitors of synoviolin have been shown to suppress histological arthritis scores in mouse RA models [38]. By contrast, synoviolin knockdown has not been associated with any impact on arthritis development [39]. It is possible that the effect of synoviolin in RA depends on its expression level. Nonetheless, monitoring synoviolin expression may help differentiate responders from non-responders to biological therapy because the higher expression has been observed in patients who did not respond to infliximab [40]. Another important stress-related mechanism involves the activation of the nod-like receptor protein 3 (NLRP3) inflammasome. It promotes the maturation of IL-1β and IL-18, thus contributing to the pro-inflammatory responses and pyroptosis [41]. Nevertheless, the involvement of NEAT1 in NLRP3 activation is complex. Current studies demonstrated that NEAT1 can both promote and suppress the inflammasome, which suggest the effect could be context-dependent [42,43,44].

As previously mentioned, lncRNA can interact with nucleic acids and proteins, enabling various mechanisms of action. In the context of RA, the most common mechanism involves the sponging of miRNA molecules, thereby reversing the inhibitory function of miRNAs and enhancing the translation process. Notably, ncRNAs interact with a range of other molecules, forming a complex network of interactions, which makes it challenging to identify the most critical signaling axis. The activity of specific pathways may depend on cellular context or environmental stimuli. In terms of NEAT1’s pro-inflammatory effects, targeting miRNA-204-5p has been identified as a mechanism of action [17]. In another study, NEAT1 was found to mediate IL-18 expression through the histone acetyltransferase p300 [26]. These enzymes are part of the epigenetic regulatory mechanisms that control gene expression. Similarly to ncRNAs, a single mechanism or enzyme can influence the expression of numerous molecules. A previous study suggested that inhibiting p300 could suppress inflammatory responses in an RA mouse model [45]. However, the role of p300 in RA inflammatory pathways is complex. Krosel et al. [46] demonstrated that exposure of synovial fibroblasts to TNF-α reduces p300 expression by approximately 50%, which affects stress response pathways alongside TNF-associated pro-inflammatory responses. It is possible that the influence of p300 on inflammatory pathways depends on its expression level or availability. The use of a histone acetyltransferase inhibitor has been shown to reduce NF-κB transcription factor activity in RA synovial cells [47]. These studies underscore the intricate role of epigenetics within the inflammatory landscape of RA. NEAT1 is significantly involved in these mechanisms, mediating its biological effects through multiple pathways. Importantly, an in vivo experiment showed that NEAT1 knockdown reduces arthritis scores [32], making NEAT1 a promising potential therapeutic target.

HOX transcript antisense RNA (HOTAIR) is another lncRNA molecule implicated in inflammatory responses. The expression of HOTAIR in PBMCs and its serum levels are elevated in patients with RA [48,49]. Its expression correlates with clinical and diagnostic parameters; for example, positive associations have been found between HOTAIR and matrix metalloproteinase 9 (MMP-9) and ESR levels [49]. In a study by Medhat et al. [50], higher HOTAIR expression was observed in patients with shorter morning stiffness (<30 min), shorter disease duration (<5 years), and the presence of rheumatoid factor (RF). Conversely, HOTAIR expression in RA-FLS has been found to be decreased [51]. This molecule has an interesting inflammatory mechanism involving macrophages, as HOTAIR presence in exosomes stimulates macrophage migration [48]. The M1 macrophages are a major cell population contributing to the RA phenotype through the secretion of TNF-α and other pro-inflammatory cytokines.

Interestingly, HOTAIR shows an expression pattern linked to other epigenetic mechanisms. Specifically, Elhai et al. demonstrated that HOTAIR is not expressed in hand joints, where they observed a greater presence of suppressive H3K27me3 histone methylation. Notably, HOTAIR expression was higher in patients with osteoarthritis (OA) than in those with RA. Additionally, stimulation of FLSs with pro-inflammatory mediators downregulated HOTAIR expression, while silencing HOTAIR was associated with almost 8000 differentially expressed genes [52].

Furthermore, the lncRNA HOTAIR has recently been shown to affect endothelial cells, whose role in RA pathogenesis has been widely studied. Endothelial cells are involved in angiogenesis, which promotes immune cell infiltration. A recent in vitro study showed that co-culturing endothelial cells with RA-FLSs overexpressing HOTAIR significantly increased the ability of human umbilical vein endothelial cells to migrate and form tubes [53], suggesting enhanced angiogenesis. In another study, endothelial cells stimulated with uric acid highlighted the potential involvement of HOTAIR in NLRP3 inflammasome activation and pyroptosis [54]. Similar findings were observed in retinal endothelial cells in the context of diabetic retinopathy [55]. To the best of our knowledge, no studies have investigated the possible relationship between HOTAIR and NLRP3 in RA.

In macrophages, HOTAIR expression is NF-κB-dependent, with NF-κB inhibitors downregulating its expression when cells are stimulated with lipopolysaccharide, a TLR stimulant. Additionally, HOTAIR is associated with the pro-inflammatory responses of lipopolysaccharide, which induced the expression of this lncRNA. Conversely, its knockdown reduces the secretion of pro-inflammatory cytokines [56]. In RA, other TLR enhancers, such as high mobility group box 1 (HMGB1), are also present [57]. The presence of molecules that interact with TLRs and stimulate NF-κB activity may be associated with increased HOTAIR expression in cell types other than FLSs, thereby promoting inflammatory responses. However, the interaction between HOTAIR and NF-κB appears complex, with conflicting findings. While some studies demonstrate that HOTAIR suppresses NF-κB activity [58], others suggest that it can stimulate its function. In macrophages stimulated with oxidized low-density lipoprotein (an atherosclerosis model), elevated HOTAIR expression reduces NF-κB expression [59]. By contrast, HOTAIR upregulated NF-κB in oncological models [60,61,62]. Unfortunately, the paper examining this issue in the RA model was retracted. These complex regulatory mechanisms between HOTAIR and NF-κB could be context-dependent. Furthermore, they could represent feedback loop mechanisms. In the classic negative feedback loop, the lncRNA could suppress the activity of the upstream mediator. However, under pathological conditions, these mechanisms could be altered, which would change the interactions between lncRNA and pro-inflammatory transcription factors.

**Figure 2:**
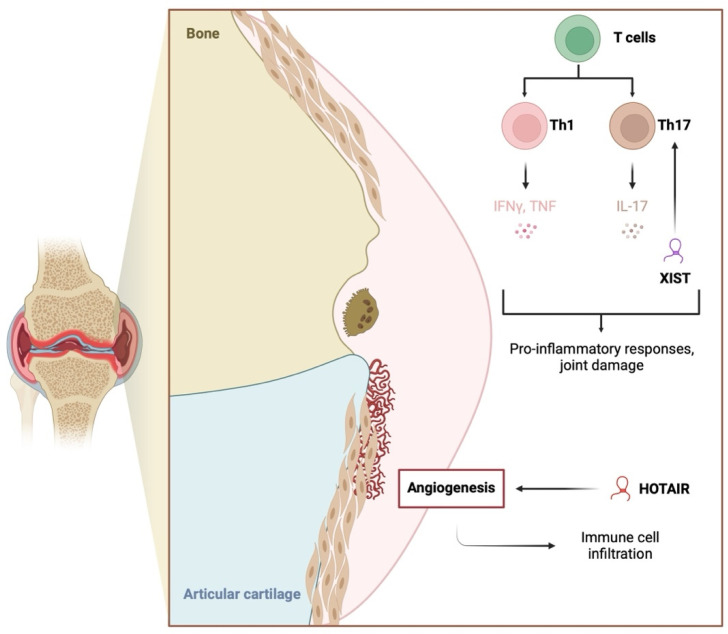
The involvement of the long non-coding RNA molecules HOTAIR and XIST on inflammation and conditions indirectly associated with inflammatory responses. HOTAIR promotes angiogenesis, which then stimulates immune cell infiltration. XIST stimulates the expression of the YY1 transcription factor that enhances the secretion of pro-inflammatory mediators and Th17 cell differentiation. Created in BioRender. Kiełbowski, K. (2025) https://BioRender.com/k18y324.

The lncRNA X-inactive specific transcript (XIST) is another molecule likely associated with inflammation and RA. The introduction of si-XIST in RA-FLSs downregulates elements of the NF-κB pathway [63]. XIST expression is elevated in the synovium of animal RA models and is linked to the expression of the Yin Yang 1 (YY1) transcription factor [64]. YY1 is thought to play an important role in RA pathogenesis because it stimulates elements of the JAK/STAT pathway and IL-6, which contribute to the chronic inflammatory environment in RA joints [65]. Additionally, YY1 promotes Th17 differentiation [66]. Its expression is also mediated by NF-κB and other pro-inflammatory downstream elements [67]. A brief summary of the roles of NEAT1, HOTAIR, and XIST in RA inflammatory responses is presented in Figure 2. The most recent research demonstrates other mechanisms linking lncRNA and NF-κB. Sun et al. showed that lnc-AL928768.3 is upregulated in RA-FLSs and that it can promote inflammatory responses by inducing lymphotoxin beta (LTB)-mediated NF-κB signaling. LTB is a member of the TNF cytokine family, and its expression has been associated with the expression of pro-inflammatory mediators [68]. Recently, the expression of lncRNA SNHG3 was found to be reduced in the serum of RA patients. In an in vivo rat model, upregulation of SNHG3 reduced inflammation and oxidative stress, thus demonstrating the involvement of this molecule in immunoregulatory pathways in RA [69].

### 2.2. RA-FLS Functionality

Various lncRNA molecules regulate inflammatory responses and RA-FLS behavior. Their aberrant expression contributes to the pathogenesis of RA. In the case of FLSs, differentially expressed molecules contribute to abnormal proliferation, migration, invasion, and resistance to apoptosis. These processes collectively lead to synovial hyperplasia, joint destruction, and persistent inflammation in RA. By modulating gene expression through interactions with proteins and miRNAs, lncRNAs influence the pathological aggressiveness of FLSs, making them potential therapeutic targets for RA treatment [16,70,71,72,73].

The aberrant proliferation of RA-FLSs is a hallmark of synovial hyperplasia in RA. This excessive cell growth leads to the formation of pannus, a thickened layer of inflamed synovial tissue that invades and destroys cartilage and bone. Numerous studies have shown that specific lncRNAs contribute to the dysregulation of cell proliferation in RA-FLSs [16,70,71]. One such lncRNA is LncNFYB, which promotes RA-FLS proliferation via the LncNFYB/ANXA2/ERK1/2 axis. In RA, LncNFYB is upregulated, as demonstrated in comparative studies between RA-FLSs and OA-FLSs. This lncRNA binds to ANXA2, a protein involved in various cellular processes, which becomes phosphorylated at Tyr24, activating the ERK1/2 signaling pathway and promoting RA-FLS proliferation. Knockdown of LncNFYB decreases FLS proliferation, while overexpression increases it, identifying LncNFYB as a potential therapeutic target for controlling FLS hyperplasia in RA [74].

Other lncRNAs, such as LINC00152 and LINC00665, also promote FLS proliferation by interacting with key regulatory proteins, including transcription factors and signaling molecules. These lncRNAs activate pathways such as PI3K/AKT and NF-κB, which are associated with cell cycle progression and survival, further enhancing FLS proliferation and contributing to the aggressive nature of RA synovial tissue [75,76,77].

lncRNAs can modulate FLS proliferation either positively or negatively [16,70,71]. For instance, LERFS negatively regulates FLS proliferation by inhibiting pro-inflammatory signaling pathways [72], while NEAT1 promotes FLS proliferation by modulating the miR-410-3p/YY1 signaling axis. Recent findings show that NEAT1 is upregulated in synovial tissues and FLSs derived from patients with RA. This overexpression is closely linked to the aggressive phenotype of RA-FLSs, characterized by increased proliferation, migration, and invasion. NEAT1 overexpression promotes the S-to-G2/M phase transition, a critical step in cell cycle progression, leading to increased FLS proliferation. By contrast, NEAT1 knockdown induces cell cycle arrest and significantly reduces RA-FLS proliferation. In addition to promoting cell growth, NEAT1 has an anti-apoptotic effect; its upregulation suppresses programmed cell death in RA-FLSs, contributing to their persistence in synovial tissue. Elevated NEAT1 levels likely contribute to the hyperplasia and synovial aggression characteristic of RA, underscoring NEAT1’s crucial role in disease progression [70,78,79,80,81].

The previously mentioned cytoplasmic lncRNA LERFS not only negatively regulates proliferation but also inhibits migration and invasion. In healthy FLSs, LERFS binds to hnRNP Q to form a complex that stabilizes or reduces the translation of key mRNAs associated with cell motility, including RhoA, Rac1, and CDC42—small GTPase proteins that regulate FLS motility and proliferation. In RA-FLSs, decreased LERFS levels reduce the formation of the LERFS–hnRNP Q complex, leading to the increased stability and translation of these target mRNAs, promoting enhanced FLS migration, invasion, and proliferation. This imbalance contributes to synovial hyperplasia and joint destruction in RA. Overexpression of LERFS in RA-FLSs has been shown to reverse these effects, suggesting that restoring LERFS levels could serve as a therapeutic strategy to reduce FLS-driven joint damage [72,81].

OSER1-AS1 is another lncRNA identified as a key regulator in RA. Its expression is significantly reduced in RA-FLSs. Overexpression of OSER1-AS1 inhibits RA-FLS proliferation and promotes apoptosis by sponging miR-1298-5p, an miRNA that targets the E2F1 transcription factor involved in cell cycle regulation. By modulating the OSER1-AS1/miR-1298-5p/E2F1 axis, OSER1-AS1 reduces pathological RA-FLS proliferation and enhances apoptosis, thereby decreasing synovial inflammation and joint damage. This indicates that OSER1-AS1 could be a valuable therapeutic target for controlling FLS hyperplasia in RA [78].

Other lncRNAs include RP11-83J16.1, which promotes FLS proliferation, migration, and inflammation, thereby exacerbating RA pathology and DILC, which can suppress FLS proliferation and reduce IL-6 expression [82,83]. By contrast, GAS5 inhibits FLS proliferation by modulating the miR-128-3p/HDAC4 axis, which controls inflammatory responses in RA [73,84]. Another lncRNA, NR-133666, also promotes FLS proliferation in collagen-induced arthritis, a model for RA. NR-133666 acts as a competing endogenous RNA, sponging miR-133c, which normally inhibits the MAPK1 gene. By reducing miR-133c’s inhibitory effect, NR-133666 increases MAPK1 expression, activating the MAPK/ERK signaling pathway and driving FLS proliferation [85].

The invasive and migratory characteristics of RA-FLSs are critical to the destructive nature of RA. RA-FLSs migrate from the synovial membrane into adjacent cartilage and bone, contributing to joint erosion and destruction. Several lncRNAs are linked to the enhanced migration and invasion of RA-FLSs [16,70,71,84]. For instance, HOTAIR promotes FLS migration and invasion by regulating the expression of MMPs, particularly MMP-2 and MMP-9. These enzymes degrade extracellular matrix components, facilitating the invasive potential of RA-FLSs. HOTAIR upregulates MMP expression through the Wnt/β-catenin pathway, a well-known regulator of cell migration and invasion in various diseases, including cancer. Silencing HOTAIR in RA-FLSs reduces migration and invasion, suggesting that targeting this lncRNA could help mitigate joint damage in RA [52].

Similarly, the lncRNA MALAT1 plays a key role in promoting RA-FLS migration. MALAT1 activates the PI3K/AKT signaling pathway, which enhances cytoskeletal rearrangement and cell motility. Increased MALAT1 expression correlates with higher FLS migration rates, while MALAT1 knockdown significantly reduces FLS invasiveness [86]. lncRNA HAFML enhances FLS migration and invasion by stabilizing the mRNA of APPL2 through interaction with the RNA-binding protein HuR, promoting mRNA expression and facilitating FLS invasion. HAFML exhibits increased expression in FLSs and synovial tissues from patients with RA [80]. Likewise, GAPLINC and PICSAR act as miRNA sponges, sequestering miRNAs that would otherwise suppress RA-FLS migration and invasion. These findings suggest that targeting lncRNAs involved in migration could inhibit aggressive synovial behavior in RA [24]. In contrast, LERFS is downregulated in RA and functions as a negative regulator of FLS migration and invasion. Overexpression of LERFS in RA-FLSs reduces their aggressive behavior [72]. Similarly to its role in proliferation, NR-133666 enhances FLS migration by modulating the miR-133c/MAPK1 axis. NR-133666 overexpression leads to elevated MAPK1 levels, which activate the MAPK/ERK signaling pathway, closely linked to cell migration in RA. Inhibiting NR-133666 reduces FLS migration, indicating its potential as a therapeutic target to prevent joint invasion [85].

In addition to its role in cell growth, NEAT1 also influences the migratory and invasive properties of RA-FLSs. NEAT1 upregulation enhances the ability of RA-FLSs to migrate and invade surrounding tissues, contributing to the destructive synovitis characteristic of RA, likely through interactions with signaling pathways controlling cytoskeletal dynamics and cell adhesion [79]. Other lncRNAs, such as ZFAS1 and H19, are also implicated in promoting FLS migration and invasion, further highlighting the diverse regulatory roles of lncRNAs in RA pathology. For example, ZFAS1 has been shown to influence RA-FLS migration and invasion through its interaction with miR-27a [87].

Dysregulation of apoptosis, or programmed cell death, contributes to the accumulation of cells in the synovial lining. In healthy synovial tissue, FLSs undergo apoptosis to maintain tissue homeostasis. However, in RA, these cells exhibit resistance to apoptosis, contributing to persistent synovial hyperplasia and chronic inflammation [16,70,71,84]. lncRNAs are also involved in modulating apoptosis in RA-FLSs. For example, lncRNA PVT1 has been found to inhibit apoptosis by interacting with the expression of Sirt6. Overexpression of PVT1 in RA-FLSs upregulates Bcl-2 expression, thereby preventing the activation of caspase-3 and -9, which are critical enzymes in the apoptosis pathway. This enables RA-FLSs to evade programmed cell death and continue proliferating within the synovium. Conversely, knockdown of PVT1 induces apoptosis in RA-FLSs, suggesting that PVT1 could be a promising target for restoring normal apoptotic processes in RA [88].

The aforementioned NEAT1 is also involved in apoptosis regulation. NEAT1 has been shown to inhibit FLS apoptosis by modulating the expression of the tumor suppressor protein p53. By reducing p53 activity, NEAT1 prevents the initiation of apoptosis, contributing to the prolonged survival of FLSs in the inflamed synovium [79,81]. Other lncRNAs, such as SNHG1 and HMS, have been implicated in promoting FLS survival by interacting with proteins and stabilizing anti-apoptotic mRNAs. The inhibition of these lncRNAs may enhance apoptosis in RA-FLSs, potentially reducing synovial hyperplasia and inflammation [78,80]. The previously described OSER1-AS1 not only inhibits FLS proliferation but also promotes apoptosis, reducing RA-FLS survival. Acting as a competing endogenous RNA for miR-1298-5p, OSER1-AS1 increases E2F1 levels, which leads to enhanced apoptotic signaling in RA-FLSs [78]. The lncRNA DILC, known to regulate apoptosis in liver cancer cells, has recently shown a similar role in RA. In patients with RA, DILC expression is significantly downregulated compared with healthy controls, correlating with decreased apoptosis in FLSs and allowing these cells to persist and proliferate in synovial tissue. The overexpression of DILC in RA-FLSs promotes apoptosis, suggesting that DILC may act as a negative regulator of FLS survival, potentially restoring normal apoptotic processes and reducing synovial inflammation. By promoting FLS apoptosis, DILC limits synovial hyperplasia, a key contributor to joint damage in RA [82].

In addition, altered functionality of RA-FLSs compared to healthy cells is associated with differences in glucose metabolism. It was demonstrated that glycolysis is activated in RA-FLSs [89]. lncRNAs also regulate metabolic pathways, as demonstrated by Zhang et al. [90] The authors found upregulated expression of lncRNA TUG1 in human RA-FLSs. Further analyses demonstrated that TUG1 affects the expression of lactate dehydrogenase A, a crucial enzyme mediating glucose metabolism.

LncRNA plays a critical role in regulating the aggressive behavior of FLSs in RA. Through their effects on proliferation, migration, apoptosis, and inflammation, lncRNAs such as OSER1-AS1, HAFML, LERFS, and GAS5 offer promising therapeutic targets. By modulating key signaling pathways and acting as sponges for miRNAs, these lncRNAs have the potential to control RA progression and reduce joint destruction. Collectively, these lncRNAs demonstrate the diverse mechanisms through which they influence FLS behavior, and their modulation presents promising therapeutic opportunities to address synovial hyperplasia and joint damage in RA (Table 1).

## 3. Long Non-Coding RNAs as Diagnostic Biomarkers in Rheumatoid Arthritis

The potential of monitoring plasma lncRNAs to diagnose or evaluate RA activity is an intriguing and expanding field of research. For example, the previously mentioned lnc-AL928768.3 was examined as a potential diagnostic biomarker. Sun et al. [19] performed ROC curve analyses and demonstrated that the area under the curve (AUC) of lnc-AL928768.3 was 0.752. However, the combination of lnc-AL928768.3 with lnc-AC091493.1 was associated with improved AUC to 0.827. lncRNA IFNG-AS1 is significantly upregulated in the peripheral blood of patients with RA and is associated with increased expression of IFNG, which encodes interferon, a Th1-related cytokine [91]. However, other studies have shown no substantial difference in IFNG-AS1 levels between individuals diagnosed with RA and control groups [92].

GAS5, RMRP, and THRIL are significantly elevated in the T cells of patients with RA, suggesting their potential role in the disease’s inflammatory response and their utility in distinguishing patients with RA from healthy individuals. A significant correlation was observed between RMRP levels and RA duration. The analysis also examined the levels of TNF-α and IL-17 cytokines, which were elevated in individuals diagnosed with RA; however, no association was found between these cytokines and the lncRNAs studied. The diagnostic efficacy of these lncRNAs was assessed using receiver operating characteristic (ROC) curve analysis. GAS5, RMRP, and THRIL showed promise in differentiating patients with RA from healthy individuals, whereas IFNG-AS1 lacked predictive significance. These findings suggest that GAS5, RMRP, and THRIL could serve as useful biomarkers for RA diagnosis, though further studies are necessary to fully understand their roles and clinical potential [92].

Recent research highlights the potential of lncRNA protein tyrosine phosphatase, receptor type E (PTPRE), as a diagnostic biomarker for RA, particularly for seronegative RA (SNRA). PTPRE is detectable in PBMCs and is upregulated in SNRA patients compared with healthy donors. Traditional diagnostic markers for RA, such as RF and anti-citrullinated protein antibodies (ACPA), have limitations, especially in early-stage RA and SNRA. In this context, PTPRE has emerged as a promising candidate. Studies have shown that PTPRE is upregulated in SNRA patients relative to healthy donors, suggesting its potential specificity for SNRA diagnosis. The diagnostic value of PTPRE was further supported by ROC curve analysis, demonstrating its utility in distinguishing SNRA patients from healthy individuals [93].

The integration of PTPRE into diagnostic protocols could complement existing methods, offering a more comprehensive approach to RA diagnosis. This aligns with ongoing efforts to identify and validate new biomarkers that can overcome the limitations of current diagnostic tools, ultimately improving patient outcomes through earlier and more accurate RA detection. Current studies identify PTPRE as a marker for SNRA diagnosis, suggesting that future research may explore its role in monitoring treatment responses [93,94].

The lncRNA ENST00000483588, transcribed from FAM211A-AS1, is significantly overexpressed in FLSs from patients with RA. A positive correlation has been observed between ENST00000483588 expression levels and clinical markers such as CRP and the Simplified Disease Activity Index (SDAI) score. This suggests that higher levels of this lncRNA are associated with increased disease activity and inflammation in patients with RA. Given its correlation with the CRP level and SDAI score, ENST00000483588 could potentially serve as a biomarker for assessing disease activity in RA. While the study highlights the association of ENST00000483588 with disease activity markers, it does not specifically address its potential for monitoring treatment response [95].

The combination of serum ITGB2-AS1 with intercellular adhesion molecule 1 (ICAM1) has demonstrated high diagnostic accuracy for RA, with a sensitivity of 86.05% and specificity of 91.67%. It outperforms traditional markers such as RF, CRP, and ESR and offers higher sensitivity than ACPA, making it a valuable tool in the clinical setting for RA diagnosis. Serum ITGB2-AS1 levels have been positively correlated with disease activity in patients with RA as measured by the DAS28, underscoring its potential utility not only as a diagnostic marker but also in monitoring disease progression and activity [94]. TCONS_I2_00013502 is upregulated while ENST00000363624 is downregulated in the peripheral serum exosomes of patients with RA compared with healthy individuals. The combination of these two lncRNAs with ACPA significantly improves the accuracy of RA diagnosis as shown by ROC curve analysis. This research highlights the significance of lncRNAs in serum exosomes as diagnostic biomarkers for RA. The association of TCONS_I2_00013502 with ACPA levels indicates a potential regulatory function in immune responses, which could be relevant for treatment monitoring, although not explicitly discussed [96].

NORAD is recognized for its significant diagnostic potential in RA and is prevalent in the serum of patients with RA. NORAD expression correlates positively with clinical indicators such as CRP, RF, ESR, and ACPA. While other lncRNAs like PICSAR and GAS5 have been investigated for RA, NORAD stands out for its strong correlation with clinical indicators, making it a promising biomarker for early diagnosis and monitoring of RA progression. This suggests that NORAD could potentially be used to monitor changes in these indicators during treatment [97].

Plasma levels of lnc-ITSN1-2 are significantly elevated in patients with RA compared with healthy controls, indicating its potential as a diagnostic marker. The expression of lnc-ITSN1-2 in plasma is positively associated with clinical features of RA, such as ESR, CRP, and DAS28, further supporting its role in disease activity assessment. lnc-ITSN1-2 is identified as a potential diagnostic biomarker for RA, with strong links to disease activity and diagnostic precision. While the research does not specifically address the application of lnc-ITSN1-2 for monitoring therapeutic efficacy over time, its robust association with disease activity markers suggests that future investigations could consider its utility in evaluating patient responses to RA treatments [98]. Recently, another panel of lncRNAs was suggested as diagnostic biomarkers. The AUC values of LINC00152, Lnc-ADM-1, and lnc-FTH1-7 are 0.618, 0.590, and 0.655, respectively. However, creating the panel of these three molecules with ITSN1-2 increases the AUC to 0.886 [99]. The AUC value of recently examined lncRNA SNHG3 was 0.915, demonstrating very promising potential to discriminate RA patients [69]. Table 2 summarizes the potential therapeutic and diagnostic role of selected lncRNAs.

The expression profiles of lncRNAs in plasma and serum exosomes suggest they may play roles in regulating immune responses and inflammation in RA, potentially through mechanisms such as cytokine production modulation and interactions with other molecular pathways [100,101]. Traditional diagnostic markers for RA, such as RF and ACPA, have several limitations that impact their clinical utility. RF sensitivity varies widely, from 26% to 90%, with a pooled sensitivity of 69% and specificity of 85% in meta-analyses, indicating that a significant number of RA cases may be missed if relying solely on RF. Although ACPA tests offer higher specificity (90–98%), they have low to moderate sensitivity (55–80%), meaning they may not detect all RA cases, particularly in the early stages. Furthermore, both RF and ACPA can be present in individuals with other conditions or even in healthy individuals, complicating diagnosis and potentially leading to false positives [94]. These limitations are particularly problematic in early RA, where symptoms vary and traditional markers may lack sensitivity, resulting in missed or delayed diagnoses. Furthermore, current RA classification criteria, which heavily rely on these markers, require the involvement of more than 10 joints when both RF and ACPA are negative, complicating the diagnosis of SNRA.

The need for new diagnostic tools is underscored by the fact that traditional markers do not adequately reflect inflammatory processes or disease activity in RA, as shown by the lack of correlation between certain lncRNAs and traditional inflammatory markers such as ESR and CRP [93]. These findings highlight the importance of lncRNAs in RA and their potential as non-invasive biomarkers for disease monitoring and therapeutic targeting. However, further research is necessary to fully elucidate their functional roles and validate their clinical utility in larger patient cohorts.

## 4. Targeting Long Non-Coding RNA

We have previously mentioned that treatment of RA can change the expression of lncRNA molecules. However, despite being a consequence of already established therapy, other strategies could be implemented to modulate the expression of lncRNA. Using antisense oligonucleotides (ASOs) represents one of the options to target these molecules. ASO-based therapeutics have been already designed. Nusinersen increases the production of survival motor neuron 1 to treat spinal muscular atrophy [102]. Inotersen is another drug developed to treat amyloidosis. It binds to the mRNA of transthyretin, inducing its damage [103]. In total, 13 ASO therapeutics have been approved [104]. Much less is known about using ASOs to target lncRNAs. Adewunmi et al. analyzed the use of ASO to target MALAT1 in preclinical models of triple-negative breast cancer. The therapeutic could significantly reduce the presence of lncRNA and suppress tumor progression [105]. Furthermore, ASO-MALAT1 could promote anti-oxidant signaling, which was beneficial in vascular smooth muscle cells [106]. As MALAT1 is implicated in the invasive features of FLS, the use of such a treatment strategy could provide benefits in RA. ASO-based NETAT1 inhibition was recently examined in the context of lipid droplet accumulation in microglia [107]. Nevertheless, to the best of our knowledge, ASO-based inhibition of lncRNA has not yet been explored in the context of RA. Other potential methods to target lncRNAs could implement peptide nucleic acids [108] or aptamers [109].

## 5. Conclusions

Current evidence suggests a significant role for lncRNAs in the pathogenesis of RA. These molecules profoundly influence inflammatory responses and the behavior of RA-FLSs. While certain lncRNAs, such as NEAT1, have been more extensively studied, the precise functions of many others remain largely unknown. The altered expression of lncRNAs, frequently observed in patients with RA, opens opportunities for developing novel therapeutic targets or biomarkers. While therapeutic applications may still be a distant goal, lncRNAs could soon serve as diagnostic and prognostic biomarkers. Monitoring lncRNAs in peripheral blood could aid in distinguishing patients with RA from healthy individuals, assessing disease duration, and evaluating clinical activity.

An intriguing future direction would be to investigate the impact of various therapeutics on lncRNA expression. Further studies could explore the potential of lncRNAs as biomarkers for treatment responses, paving the way for more personalized treatment strategies. This approach could ultimately improve treatment outcomes and reduce toxicities. 

## Figures and Tables

**Table 1:** A list of selected lncRNA molecules regulating the functionality of rheumatoid arthritis-fibroblast-like synoviocytes.

LncRNA	Impact on RA-FLS	References
LncNFYB	Promote proliferation	[74]
LINC00152	Promote proliferation	[75,76]
LINC00665	Promote proliferation	[77]
HOTAIR	Promote migration and invasion	[52]
PVT1	Promote proliferation and suppress apoptosis	[88]
NEATT	Promote proliferation, migration and invasionSuppress apoptosis	[70,78,79,81]
RP11-83J16.1	Promote proliferation, migration, and inflammation	[78,82]
NR-133666	Promote proliferation and migration	[85]
HAFML	Promote migration and invasion	[80]
GAPLINC	Promote proliferation, migration and invasion	[24]
PICSAR	Promote migration and invasion	[24]
ZFAS1	Promote migration and invasion	[87]
H19	Promotion migration and invasion	[87]
SNHG1	Suppress apoptosis	[78,80]
HMS	Suppress apoptosis	[78,80]
MALAT1	Promote apoptosis	[86]
LERFS	Suppress migration, invasion and proliferation	[72,81]
OSER1-AS1	Suppress proliferationPromote apoptosis	[78,81]
GAS5	Suppress proliferationPromote apoptosis	[73,84]
DILC	Promote apoptosis	[82]
THRIL	Suppress proliferation, migration and invasion	[73]

**Table 2:** A summary of the therapeutic and diagnostic potential of selected lncRNA. Information about ROC curve analysis and area under cover were used to describe diagnostic potential.

lncRNA	Therapeutic Potential	Diagnostic Potential	References
NEAT1	Reducing the expression of NEAT1 could be clinically beneficial	-	[17,26,32,35,70,78,79,80,81]
HOTAIR	Further studies are required due to conflicting results	-	[48,51,52]
XIST	Reducing the expression of XIST could be clinically beneficial	-	[63]
lnc-AL928768.3	Reducing the expression of lnc-AL928768.3 could be clinically beneficial	AUC 0.752	[68]
LncNFYB	Reducing the expression of LncNFYB could be clinically beneficial	-	[74]
LERFS	Increasing the expression of LERFS could be clinically beneficial	-	[72,81]
OSER1-AS1	Increasing the expression of OSER1-AS1 could be clinically beneficial	-	[78]
DILC	Increasing the expression of DILC could be clinically beneficial	-	[82]
RP11-83J16.1	Reducing the expression of RP11-83J16.1 could be clinically beneficial	-	[83]
MALAT1	Reducing the expression of MALAT1 could be clinically beneficial	-	[86]
ITGB2-AS1	-	AUC 0.772	[94]
lnc-ITSN1-2	-	AUC 0.898	[98]
LINC00152	-	AUC 0.618	[99]
Lnc-ADM-1	-	AUC 0.590	
Lnc-FTH1-7	-	AUC 0.655	
SNHG3	Increasing the expression of SNHG3 could be clinically beneficial	AUC 0.915	[69]

AUC—area under curve.

## Data Availability

Not applicable.
